# Radiation Exposure Reduction by Digital Variance Angiography in Lower Limb Angiography: A Randomized Controlled Trial

**DOI:** 10.3390/jcdd10050198

**Published:** 2023-04-30

**Authors:** Péter Sótonyi, Márton Berczeli, Marcell Gyánó, Péter Legeza, Zsuzsanna Mihály, Csaba Csobay-Novák, Ákos Pataki, Viktória Juhász, István Góg, Krisztián Szigeti, Szabolcs Osváth, János P. Kiss, Balázs Nemes

**Affiliations:** 1 Department of Vascular and Endovascular Surgery, Heart and Vascular Center, Semmelweis University, Városmajor utca 68, 1122 Budapest, Hungary; 2 Kinepict Health Ltd., Szilágyi Erzsébet Fasor 31, 1027 Budapest, Hungary; 3 Department of Interventional Radiology, Heart and Vascular Center, Semmelweis University, Városmajor utca 68, 1122 Budapest, Hungary; 4 Department of Vascular Surgery, Hungarian Defence Forces Medical Centre, Róbert Károly körút 44, 1134 Budapest, Hungary; 5 Department of Biophysics and Radiation Biology, Semmelweis University, Tűzoltó u. 37-47, 1094 Budapest, Hungary

**Keywords:** digital variance angiography, digital subtraction angiography, radiation reduction, radiation protection, peripheral artery disease, lower limb angiography

## Abstract

Background: digital variance angiography (DVA) provides higher image quality than digital subtraction angiography (DSA). This study investigates whether the quality reserve of DVA allows for radiation dose reduction during lower limb angiography (LLA), and compares the performance of two DVA algorithms. Methods: this prospective block-randomized controlled study enrolled 114 peripheral arterial disease patients undergoing LLA into normal dose (ND, 1.2 µGy/frame, *n* = 57) or low-dose (LD, 0.36 µGy/frame, *n* = 57) groups. DSA images were generated in both groups, DVA1 and DVA2 images were generated in the LD group. Total and DSA-related radiation dose area product (DAP) were analyzed. Image quality was assessed on a 5-grade Likert scale by six readers. Results: the total and DSA-related DAP were reduced by 38% and 61% in the LD group. The overall visual evaluation scores (median (IQR)) of LD-DSA (3.50 (1.17)) were significantly lower than the ND-DSA scores (3.83 (1.00), *p* < 0.001). There was no difference between ND-DSA and LD-DVA1 (3.83 (1.17)), but the LD-DVA2 scores were significantly higher (4.00 (0.83), *p* < 0.01). The difference between LD-DVA2 and LD-DVA1 was also significant (*p* < 0.001). Conclusions: DVA significantly reduced the total and DSA-related radiation dose in LLA, without affecting the image quality. LD-DVA2 images outperformed LD-DVA1, therefore DVA2 might be especially beneficial in lower limb interventions.

## 1. Introduction

Minimally invasive endovascular interventions play an increasing role in the treatment of a wide range of pathologies [1,2,3,4]. These procedures, however, deliver high radiation doses both to the patients and the medical staff, which in turn raises serious concerns regarding the harmful effects of ionizing radiation. A recent meta-analysis on the endovascular treatment of 17,174 peripheral artery disease (PAD) patients showed that the dose-area product (DAP) exceeded the 500 Gy*cm^2^ threshold, a radiation dosage with potential tissue injury, in 7% of all cases [5]. The high radiation dose is also an occupational hazard for the medical staff [6,7], therefore radiation reduction measures are critically important in medical imaging procedures using ionizing radiation [8,9].

The majority (60–90%) of DAP associated with endovascular interventions arises from stationary acquisitions using digital subtraction angiography (DSA) [10], therefore the reduction of DSA-related radiation dose might be an effective solution. The dose reduction, however, can lead to lower image quality, and even with proper postprocessing of DSA images the acquisition might have to be repeated to gain images with sufficient diagnostic information.

Digital variance angiography (DVA), a recently developed image processing technology might provide an effective dose management solution. DVA is based on the principles of kinetic imaging [11]. Because of its advanced algorithm, it enhances the contrast media-generated signal, but suppresses the noise, which results in higher contrast-to-noise ratio and better image quality [12,13,14,15,16]. This quality reserve can be used for radiation reduction. In a retrospective proof-of-concept study, DVA facilitated the decrease of the DSA-related radiation exposure by 70% in lower limb diagnostic angiography with non-inferior or even superior image quality compared to full-dose DSA images [17].

The primary aim of this prospective pivotal study was to investigate whether the quality reserve of DVA is suitable for the reduction of radiation exposure in everyday clinical practice, when the technology is used in real-time in the operating room during lower limb angiography. A secondary aim was to compare the performance of the original DVA1 algorithm with the new DVA2 algorithm which was developed to further improve image quality under conditions, when the background noise is higher, such as in the case of radiation reduction.

## 2. Materials and Methods

The study was approved by the Hungarian National Institute of Pharmacy and Nutrition (reference number OGYÉI/2830/2017). All study activities were in accordance with the ethical standards of the Hungarian Medical Research Council and with the 1964 Helsinki Declaration. The study protocol is available on clinicaltrials.gov (NCT04343196). Written informed consent was obtained from all participants included in the study.

### 2.1. Patient Selection

This prospective randomized controlled trial enrolled 114 patients (72.8% male, median (IQR) age 66.1 (9.3) years) undergoing diagnostic lower limb angiography between April 2020 and September 2020 at our institute. Table 1 shows the detailed demographics. Participants were selected in a consecutive manner based on the eligibility criteria. Inclusion criteria were patients with symptomatic (Fontaine IIb-IV) PAD, glomerular filtration rate over 60 mL/min/1.73 m^2^ and age over 50 years. Exclusion criteria were acute myocardial infarction, atrioventricular block, severe heart, liver or renal failure and patients with acute lower limb arterial occlusion. Enrolment ended when the planned participant number was reached. The number of patients was selected according to an FDA Guideline developed for the concurrence testing of X-ray imaging devices [18]. Patients received clinical standard of care and all procedures were performed according to the institutional protocol.

### 2.2. Study Design

**Figure 1:**
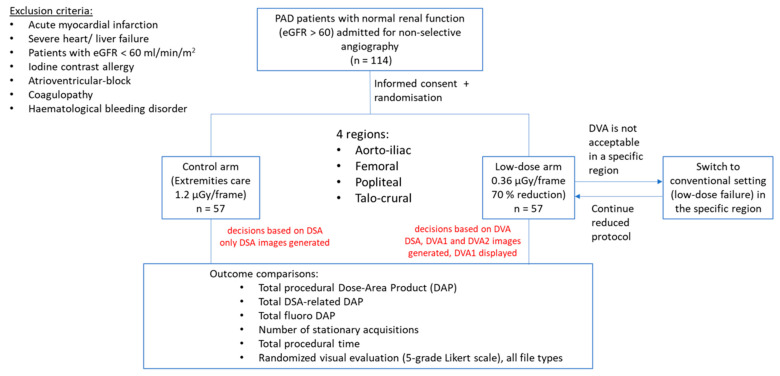
Flow chart of the study. Peripheral artery disease (PAD) patients were enrolled in a consecutive manner between April and September 2020. Patients were block-randomized into a control (normal dose) and a low-dose arm. In the low-dose arm, there was a possibility to switch back to normal dose imaging if the low-dose images were not appropriate for diagnosis (low-dose failure).

Patients were randomly assigned to normal dose (ND) or low-dose (LD) group using a randomization plan generator website (for details see the Supplementary Material). The ND group was exposed to standard radiation during stationary acquisitions, whereas the dose/frame value was reduced by 70% in the LD group. There was no other difference in acquisition parameters, the fluoroscopy settings were identical. In the ND group, only the DSA images were calculated and displayed, while in the LD group, DSA, DVA1 and DVA2 images were generated in real time, but the diagnostic decisions were based on DVA1 images. The total DAP (µGy*m^2^) and stationary acquisition/DSA-related DAP, the number of stationary acquisitions, total contrast media use and procedural time were extracted from dose-reports. Procedural time was defined as the time from arterial access to device removal. The visual evaluation of different image types was accomplished in a retrospective web-based survey. A flowchart of the study is shown in Figure 1.

### 2.3. Image Acquisition

Lower limb angiography was performed according to the institutional protocol using a Siemens Artis Zee with Pure system and a Syngo XWP VD11B SP2 workstation (Siemens Healthcare, Munich). The Siemens Extremities Care DSA protocol was used for ND (1.2 µGy/frame) and its modified version for LD (0.36 µGy/frame) imaging. A diagnostic catheter (Impulse, PIG 5F 125 cm; Boston Scientific, Marlborough, MA, USA) was introduced from radial access into the aorta, and after an infrarenal aortography (aortoiliac image at 2 FPS) it was repositioned above the aortic bifurcation. All further injections (femoral, popliteal and talocrural images at 1 FPS) occurred from this position. A Medrad Avanta automatized injector (Bayer, Indianola, PA, USA) was used for injecting 7–15 mL/injection contrast media (Ultravist 370, Bayer, Leverkusen, Germany) at 9 mL/s flowrate.

### 2.4. Image Processing

Three types of images were generated from the raw radiographic image series during the endovascular procedure, and saved as Digital Imaging and Communication in Medicine (DICOM) files. DSA images were created by the Siemens Syngo workstation in both groups (ND-DSA, LD-DSA), whereas DVA images were generated by the Kinepict Medical Imaging Tool v4.0 (Kinepict Health Ltd., Budapest, Hungary) only in the LD group (LD-DVA1 and LD-DVA2). As DVA images were generated in real-time, the interventional radiologist could see DVA1 images on the operating room monitor immediately after the image acquisition. As DVA2 images were not tested previously, they were not used for diagnosis, and were prepared only for the performance comparison with DVA1.

### 2.5. Visual Evaluation

A blind evaluation of the images was conducted by two vascular surgeons (initials followed by the years of experience: P.S. 23, V.J. 7) and four interventional radiologists (B.N. 30, C.N. 9, A.P. 11, M.G. 7). DSA and DVA images were evaluated by the six readers using a 5-grade rating scale ranging from poor (1) to outstanding (5) image quality (for details see table legends). The rating scale was implemented in a blind and randomized web-based questionnaire, and the data were collected automatically in a database for later processing.

### 2.6. Statistical Analysis

Categorical data are presented as number (%), continuous data are presented as mean ± SEM, and because of the non-Gaussian distribution, as median (interquartile range). Normal distribution was assessed by the Shapiro-Wilk test of normality. Differences between categorical data was assessed by chi-square test or the two-sided Z-test. DAP values were compared using the Mann-Whitney U test, visual scores were compared using the Kruskal-Wallis test followed by Dunn test (ND-DSA vs. LD file types) or by the Wilcoxon signed rank test. Kendall W was used to characterize the interrater agreement. The results were considered significant at *p* < 0.05. SPSS (IBM Corp Armonk, NY, USA) and Prism 8.4 (GraphPad, San Diego, CA, USA) were used for statistical analysis.

## 3. Results

### 3.1. Baseline Characteristics and Procedural Details

A total number of 114 PAD patients were included (72.8% male, median (IQR) age 66 (9.25) years). Patient characteristics are presented in Table 1. The number of stationary (DSA) acquisitions did not differ significantly between the ND and LD groups (median (IQR) 6 (2) vs. 6 (2), *p* = 0.41). There was no significant difference in the median contrast use (79 (26) vs. 87 (26) mL, *p* = 0.13) or in the procedural time (9.5 (6.0) min vs. 10.0 (5.5) min, *p* = 0.93) (Table 2). Low-dose protocol failure, i.e., the need for switching back to the normal dose because of the non-diagnostic image quality (Figure 1), did not occur in the study.

### 3.2. Radiation Dose

**Figure 2:**
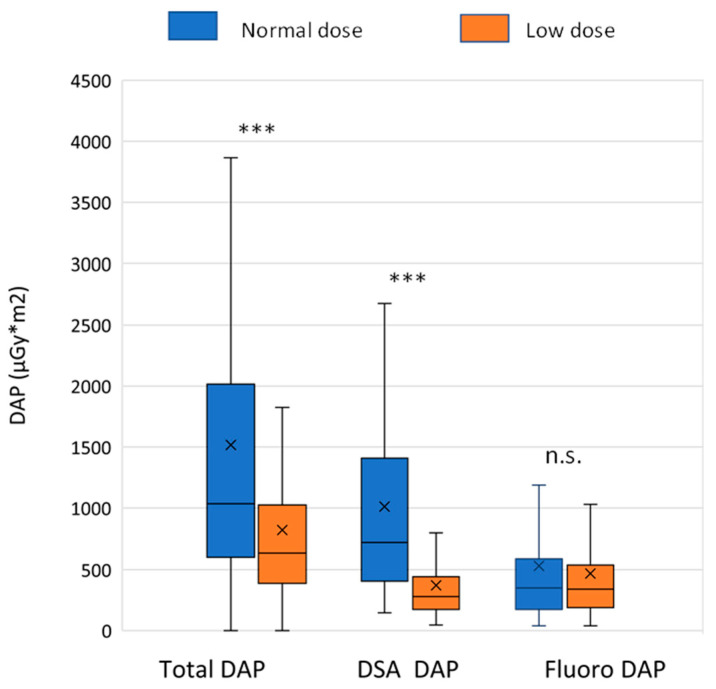
Radiation dose data. Dose-area product (DAP) values were extracted from the dose reports. The box and whisker plots show the median (line), interquartile range (box) and internal fences (whiskers), x denotes the mean value. The Mann-Whitney test was used for statistical analysis (*** *p* < 0.001). DSA: digital subtraction angiography.

The total DAP (median (IQR)) during angiographic procedures was significantly lower in the LD group compared to ND group (642.3 (614.9) vs. 1044.8 (1417.3) µGy*m^2^, Mann-Whitney U test *p* < 0.001). Similarly, the stationary acquisition-related DAP was significantly lower in the LD group (279.3 (268.1) vs. 724 (1002.6) µGy*m^2^, *p*< 0.001) (Figure 2). Thus, the radiation dose reduction was 38% and 61% in the median total and the DSA-related DAP, respectively, while the mean total and DSA-related reduction was even higher, 46% and 63%, respectively (Table 2). Fluoroscopy-related DAP did not differ significantly between the ND and LD groups (349.9 (414.9) vs. 340 (349.7) µGy*m^2^, *p* = 0.85).

### 3.3. Visual Evaluation

**Figure 3:**
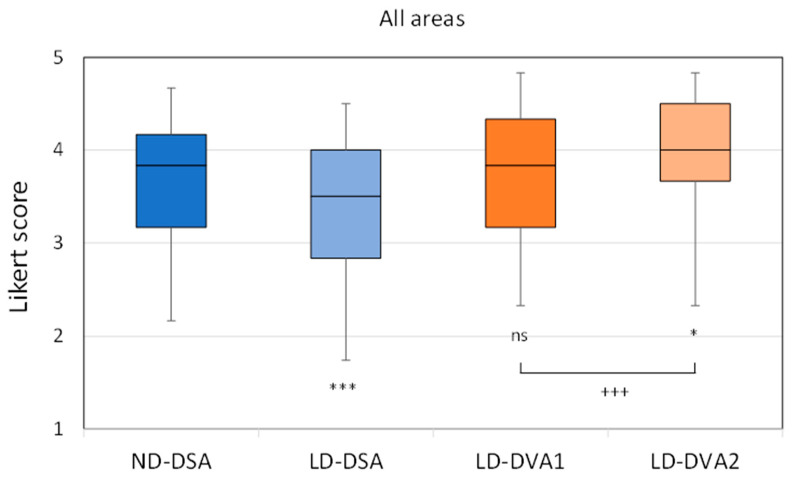
Visual evaluation data, overall results. Images were evaluated in a blind, randomized manner by six readers using a 5-grade Likert scale. All LD images were generated from the same unsubtracted series. The box and whisker plots show the median (line), interquartile range (box) and internal fences (whiskers). The Kruskal-Wallis test followed by Dunn test (* *p* < 0.05, *** *p* < 0.001) was used to compare LD groups to the ND group, whereas the Wilcoxon signed rank test (+++ *p* < 0.001) was used to compare the DVA image types. DSA: digital subtraction angiography; DVA: digital variance angiography.

Readers evaluated 557 stationary acquisitions (261 in the ND and 296 in the LD group) from four anatomical regions (aortoiliac, femoral, popliteal, talocrural). In the first analysis the LD images were compared to ND images. Overall, LD-DSA images received significantly lower scores (median (IQR) 3.50 (1.17)) than ND-DSA images (3.83 (1.00), Kruskal-Wallis followed by Dunn test *p* < 0.001). The LD-DVA1 images provided the same image quality (3.83 (1.17) as ND-DSA images, but the LD-DVA2 images received significantly higher scores (4.00 (0.83), *p* < 0.05) (Figure 3). A similar pattern could be observed in the distinct anatomical regions (Figure S1). LD-DSA provided significantly lower image quality in all regions, except in the popliteal region. LD-DVA1 was almost identical in all region with the ND-DSA images without any significant difference, and LD-DVA2 had consistently higher scores than ND-DSA (except in the aortoiliac region), but the difference was significant only in the popliteal region. For further details see Table 3. The Kendall W analysis showed a moderate to strong interrater agreement being significant (*p* < 0.001) in all regions, image types and protocol groups, The W value ranged between 0.38 and 0.48 in the aortoiliac, 0.25 and 0.51 in the femoral, 0.54 and 0.64 in the popliteal and 0.58 and 0.69 in the talocrural region (Table S1).

**Figure 4:**
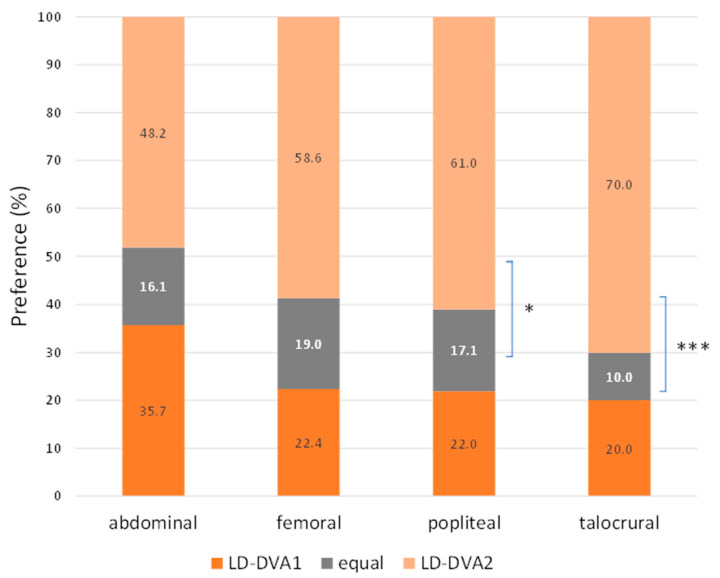
Comparison of DVA1 and DVA2 algorithms. The average Likert scores were compared in four anatomical regions. The percentage of ‘DVA2 is superior to DVA1’ cases was compared to the percentage of ‘DVA2 is not superior (equal or inferior) to DVA1’ cases using two-sided Z test (* *p* < 0.05, *** *p*< 0.001). DVA: digital variance angiography.

**Figure 5:**
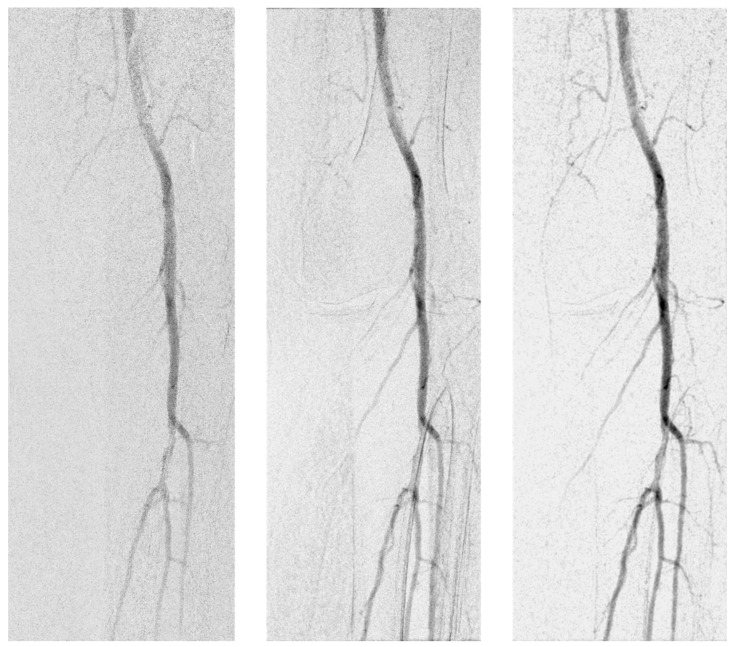
Comparison of low-dose images (left DSA, middle DVA1 right DVA2). Images were generated from the same unsubtracted series acquired in the popliteal region.

In comparison of DVA1 and DVA2 algorithms, the Wilcoxon signed rank test was used for statistical analysis, as the images pairs were generated from the same unsubtracted series. Overall, LD-DVA2 images received significantly higher scores than LD-DVA1 images (4.00 (0.83) vs. 3.83 (1.17), *p* < 0.001). Concerning the anatomical regions, no significant difference was seen between LD-DVA1 and LD-DVA2 in the aortoiliac region (4.33 (0.54) vs. 4.33 (0.5), *p* = 0.55), but LD-DVA2 received significantly higher scores in the femoral (4.17 (0.79) vs. 4.33 (0.5), *p* < 0.001), popliteal (3.67 (0.87) vs. 4.00 (0.79), *p* < 0.001) and talocrural (3.17 (1.00) vs. (3.67 (1.00), *p* < 0.001) regions compared to LD-DVA1 images (Figure S1). This gradually increasing difference was even more obvious when the average visual scores were compared (Figure 4). The average score of LD-DVA2 images was higher in 48%, 59%, 61% and 70% of image pairs in the aortoiliac, femoral, popliteal, and talocrural regions, respectively. In the latter two cases, the difference was already significant by using the two-tailed Z test (*p* < 0.05 in the popliteal and *p* < 0.001 in the talocrural region). A representative image set illustrates the difference between LD-DSA, LD-DVA1 and LD-DVA2 (Figure 5).

## 4. Discussion

The major aim of this study was to investigate whether the use of DVA technology in lower limb diagnostic angiography allows for a radiation dose reduction in clinical practice. Our data show that a 70% reduction of the dose/frame parameter in the low-dose DVA group resulted in around a 40% reduction in the total procedural dose and more than a 60% reduction in the DSA-related radiation exposure without compromising the diagnostic work. The low-dose DVA image quality was always sufficient to make diagnostic judgements, as the interventional radiologists could complete the examination without switching back to normal dose in the low-dose arm (zero low-dose failure). The retrospective visual evaluation confirmed that the low-dose acquisition provided non-inferior or superior DVA image quality in all anatomical regions compared to normal dose DSA images. An additional aim was to compare the performance of two DVA algorithms. DVA1 is the original method, while DVA2 has a built-in noise filter, therefore DVA2 can further improve the image quality, especially when the radiation dose is lower, and the obtained image tends to be noisier. Indeed, DVA2 provided identical or better image quality than DVA1. The more distal anatomical region was investigated, the higher difference could be observed between these images, and the advantage of DVA2 was significant in the popliteal and talocrural regions.

Our data have clinical significance. The reduction of radiation exposure is important not only for the patients [8,19] but also for the medical staff [9]. Apart from appropriate shielding instruments and limited working hours for staff [20], the aspects of the As Low As Reasonably Achievable (ALARA) principle are also associated with radiation dose management measures during image acquisition [21,22]. The DVA technology might provide an additional new dose management tool. DVA allowed for a 50% reduction of contrast media without compromising the image quality in carotid angiography [23]. In another study, a 70% reduction of the nominal DSA-related radiation dose was reported in lower limb angiography, while the image quality of DVA was non-inferior in the abdominal and femoral region and superior in the crural region compared to full dose DSA images [17]. Those proof-of-concept studies, however, were conducted on small cohorts and were retrospective in nature. This pivotal study enrolled 114 patients undergoing diagnostic lower limb angiography. The DVA technology was installed in the operating room and was used in real-time [24] for the diagnostic work, so this is the first report on the dose management capability of DVA in a prospective randomized controlled trial. The data clearly show that DVA allows for a very substantial reduction of total procedural and DSA-related radiation exposure, thereby it can increase the safety of endovascular lower limb procedures. As the dose management capability of DVA is based on its unique quality reserve, the technology can be used in combination with any other dose management solutions and provides an additional radiation reduction opportunity.

While CT angiography, MR angiography, duplex-ultrasound (DUS), and contrast-enhanced DUS are available in most vascular centers, diagnostic angiography is still a widely used imaging technique, due to its rapid intraoperative availability, and its high diagnostic potential mainly in the assessment of infrainguinal and below-the-knee pathology [25]. The chronic limb-threatening ischemia (CLTI) guidelines suggest obtaining high-quality angiographic imaging of the lower limb, which should include the ankle and foot in all patients with suspected CLTI [26]. Our data show that DVA2 provides outstanding image quality in these regions even with a reduced radiation dose, therefore it could help to define the target vessel pathway in below-the-knee procedures, which is essential to estimate the chance of limb salvage.

The study has some limitations. We focused only on non-selective lower limb diagnostic angiography, because this procedure is more standardized, the number of stationary acquisitions is relatively constant, therefore the comparison between the control and experimental group is more reliable. Nevertheless, a recent study suggests that the quality advantage of DVA can also be observed in lower limb interventions, where selective angiography is applied [15]. The quality reserve of DVA was also demonstrated in carotid angiography [23] and prostatic artery embolization [16], and liver transarterial chemoembolization [27]. These observations suggest that the dose management would be possible also in these endovascular interventions, but the results cannot be generalized without further investigations. The achievable radiation reduction might also depend on the type of angiography instrument and the applied standard protocol; therefore, the reported reduction values cannot be transferred automatically. The study analyzed only the dose reports, the radiation exposure of the medical staff was not measured, thus the effective patient dose was not calculated, but it can be assumed that it was also proportionally lower.

## 5. Conclusions

Our study demonstrated that the quality reserve of DVA can be used for radiation exposure reduction in the clinical practice, without compromising the image quality and diagnostic value of angiograms. The recently developed DVA2 algorithm outperformed the classical DVA1 algorithm in the popliteal and talocrural regions, therefore it might be especially useful in below-the-knee interventions. Our results warrant further studies on the dose management capability of DVA in other types of endovascular interventions.

## Figures and Tables

**Table 1:** Patient characteristics. Categorical variables are presented as n (%), and the chi-square test was used to compare the groups. Continuous variables are presented as median (IQR), and the Mann-Whitney test was used to compare the normal dose (ND) and low-dose (LD) groups. BMI: body mass index; GFR: glomerular filtration rate; COPD: chronic obstructive pulmonary disease.

	Total, Median (IQR) or n(%)	ND	LD	*p* (ND vs. LD)
Male	83 (72.8)	38 (66.6)	45 (88.9)	0.14
Age (years)	66 (9)	68 (9)	65 (11)	0.42
BMI (kg/m^2^)	26.1 (7.2)	25 (8)	26.6 (6.5)	0.35
GFR (mL/min/1.73 m^2^)	87 (16)	87 (13)	85 (19)	0.28
Smoking	43 (38)	22 (39)	21 (37)	0.91
Hypertension	82 (72)	41 (72)	41 (72)	1.00
Diabetes mellitus	51 (45)	25 (44)	26 (46)	0.84
COPD	16 (14)	9 (16)	7 (12)	0.58
Ischemic heart disease	38 (33)	16 (28)	22 (39)	0.21
Cerebrovascular disease	10 (9)	6 (11)	4 (7)	0.50
Fontaine-stage				
IIB	58 (51)	28 (49)	30 (53)	0.43
III	3 (3)	2 (4)	1 (2)	0.50
IV	53 (47)	27 (47)	26 (46)	0.50

**Table 2:** Radiation related data. Data is presented as median (IQR) or mean ± SD. Mann-Whitney U test was used to compare non-normally distributed continuous variables. Abbreviations: DAP, dose-area product, ND, normal dose, LD, low-dose.

		ND	LD	LD/ND (%)	*p*
Number of DSA acquisitions		6 (2)	6 (2)	100	0.41
Total DAP (µGy*m^2^)	Median	1044.8 (1417.3)	642.3 (614.9)	62	<0.0001
	Mean	1544.2 ± 1172.2	841.0 ± 633.9	54	
DSA-DAP (µGy*m^2^)	Median	724.2 (1002.6)	279.3 (268.1)	39	<0.0001
	Mean	1012.3 ± 790.1	372.5 ± 333.1	37	
Fluoro-DAP (µGy*m^2^)	Median	349.9 (414.9)	340.3 (349.7)	97	0.85
	Mean	532.0 ± 556.6	468.4 ± 381.9	88	
Contrast use (mL)		79 (26)	87 (26)	110	0.13
Procedural time (min)		9.5 (6.0)	10.0 (5.5)	105	0.93

**Table 3:** Results of visual evaluation. The following 5-grade Likert scale was used: (1) poor image quality, unsuitable for diagnosis; (2) low image quality, main vessels are distinguishable but not examinable; (3) medium image quality, sufficient for diagnosis in the main arteries, but smaller vessels and collateralization are not examinable; (4) good image quality, both smaller and the main vessels are examinable, suitable for everyday use; (5) outstanding image quality, much richer in details compared to the everyday routine, making decision-making easier. Data are presented as median (IQR) and mean ± SEM. The Kruskal-Wallis test was used for analyzing the complete dataset, and all groups were compared to the ND-DSA group as a control using Dunn test for multiple comparisons. When LD-DVA1 and LD-DVA2 were compared, the Wilcoxon signed rank test was used. In all cases, *p* < 0.05 was considered significant. ND: standard dose; LD: low-dose; DSA: digital subtraction angiography; DVA digital variance angiography.

		ND-DSA	LD-DSA	LD-DVA1	LD-DVA2	*p* (DVA1 vs. DVA2)
All	Median(SQR)	3.83 (1.00)	3.50 (1.17)	3.83 (1.17)	4.00 (0.83)	<0.001
	*p* (vs ND-DSA)		<0.001	>0.999	0.028	
	Mean ± SEM	3.68 ± 0.05	3.35 ± 0.05	3.69 ± 0.05	3.89 ± 0.04	
Aortoiliac	Median(SQR)	4.33 (0.66)	4.00 (0.50)	4.33 (0.54)	4.33 (0.50)	0.55
	*p* (vs ND-DSA)		0.118	>0.999	>0.999	
	Mean ± SEM	4.20 ± 0.07	3.94 ± 0.06	4.21 ± 0206	4.24 ± 0.06	
Femoral	Median(SQR)	4.16 (0.5)	3.83 (0.66)	4.17 (0.79)	4.33 (0.50)	<0.001
	*p* (vs ND-DSA)		<0.001	>0.9999	0.2024	
	Mean ± SEM	4.19 ± 0.05	3.79 ± 0.07	4.18 ± 0.06	4.35 ± 0.04	
Popliteal	Median(SQR)	3.58 (1)	3.17 (1.16)	3.67 (0.87)	4.00 (0.79)	<0.001
	*p* (vs ND-DSA)		0.2711	0.1191	<0.001	
	Mean ± SEM	3.41 ± 0.09	3.19 ± 0.09	3.67 ± 0.08	3.89 ± 0.08	
Talocrural	Median(SQR)	3.41 (1.00)	2.83 (1.00)	3.17 (1.00)	3.67 (1.00)	<0.001
	*p* (vs ND-DSA)		<0.001	0.8736		
	Mean ± SEM	3.22 ± 0.08	2.7 ± 0.10	3.13 ± 0.08	3.44 ± 0.07	

## Data Availability

The study protocol is available on clinicaltrials.gov (NCT04343196).

## Supplementary Materials

The following supporting information can be downloaded at: https://www.mdpi.com/article/10.3390/jcdd10050198/s1, Figure S1: Visual evaluation data, regional results; Table S1: Interrater agreement analysis.
