# Supplementary material for: Radiation Exposure Reduction by Digital Variance Angiography in Lower Limb Angiography: A Randomized Controlled Trial
Source: J Cardiovasc Dev Dis. 2023 Apr 30;10(5):198. doi: 10.3390/jcdd10050198 (PMC10219428; doi:10.3390/jcdd10050198)
Supplement: Supplementary file 1 [file jcdd-10-00198-s001.zip › jcdd-2322086-supplementary.pdf]

SUPPLEMENTARY MATERIAL

Randomization process

Patients were referred for the study by independent angiologists, vascular surgeons or interventional radiologists. The study team (vascular surgeon, interventional radiologist) evaluated the patient, and verified that the patient meets eligibility criteria, and has none exclusion criteria. Eligible patients, who consented and signed the informed consent were randomized.

Patients were randomly assigned to normal dose (ND) or low-dose (LD) group using a randomization plan generator website (https://www.randomizer.org). Block-randomization was used to achieve balance in the number of subjects randomized in the control (ND) and experimental (LD) groups. Randomly permuted blocks were used for randomization, with a block size of 50, except the last 14 patients who were randomized in a 14-block.

MB generated the allocation sequence using the randomization generator, GYM and MB enrolled participants and MB assigned participants to interventions based on the predetermined randomization order. The patient’s characteristics never influenced the randomization process.

Group	All	Aortoiliac	Femoral	Popliteal	Talocrural
ND-DSA	0.48	0.48	0.30	0.63	0.60
LD-DSA	0.68	0.38	0.51	0.64	0.69
LD-DVA1	0.65	0.43	0.40	0.59	0.65
LD-DVA2	0.55	0.39	0.25	0.54	0.58

Table S1. Interrater agreement analysis. The table shows the Kendall W values for the different anatomical regions and image types. The agreement was significant in every group ($p < 0.001$, except in the femoral DVA1 group where $p < 0.01$)

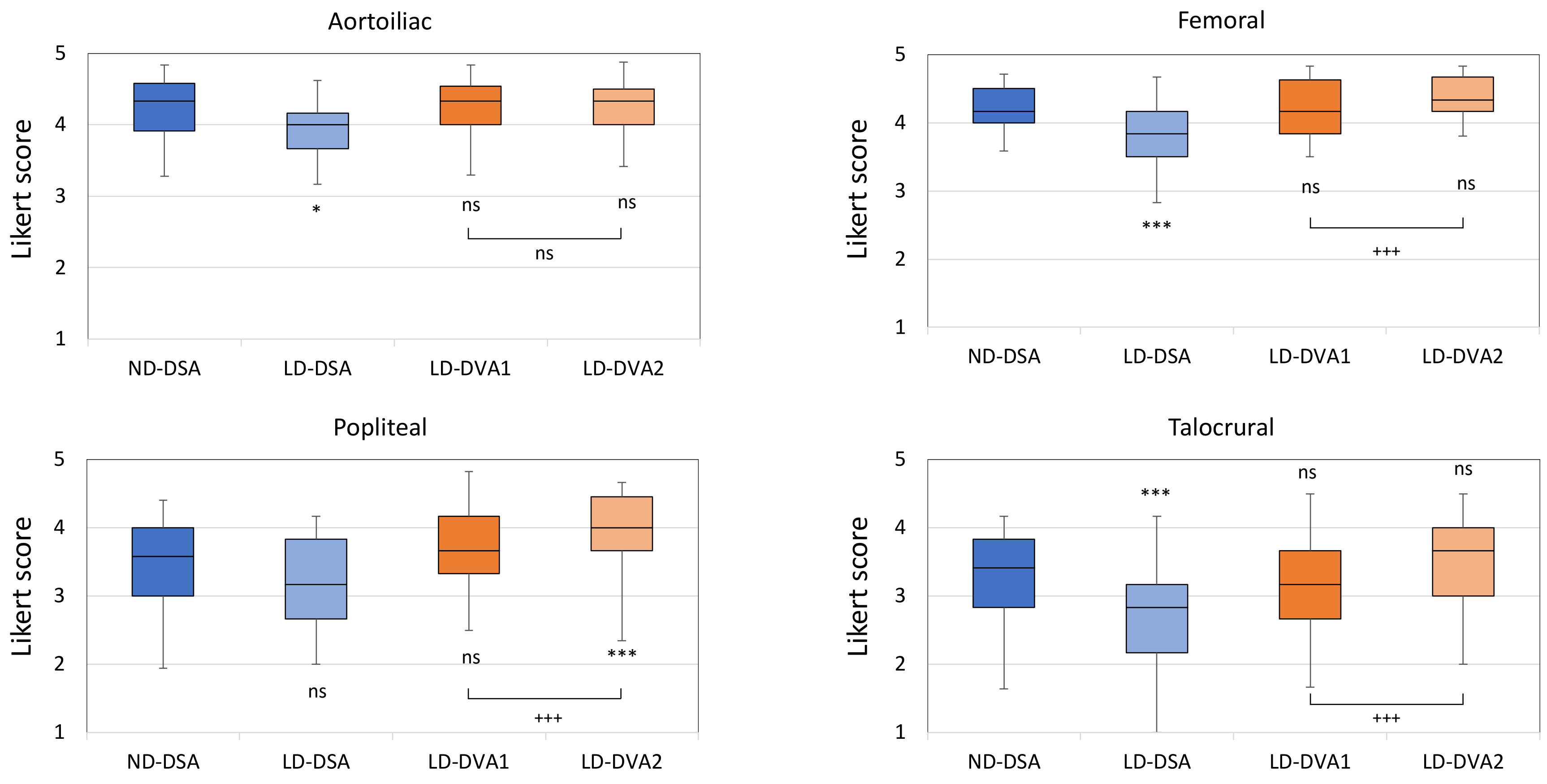

Figure S1. Visual evaluation data, regional results. Images were evaluated in a blinded, randomized manner by six readers using a 5-grade Likert scale. The LD images were generated from the same unsubtracted series. The box and whisker plots show the median (line), interquartile range (box) and internal fences (whiskers). The Kruskal Wallis test followed by Dunn test (* $p < 0.05$, *** $p < 0.001$) was used to compare LD groups to the ND group, whereas the Wilcoxon signed rank test (+++ $p < 0.001$) was used to compare the DVA image types. DSA: Digital Subtraction Angiography; DVA: Digital Variance Angiography.
